# Influenza vaccine effectiveness against laboratory-confirmed influenza in hospitalised adults aged 60 years or older, Valencia Region, Spain, 2017/18 influenza season

**DOI:** 10.2807/1560-7917.ES.2019.24.31.1800461

**Published:** 2019-08-01

**Authors:** Ainara Mira-Iglesias, F Xavier López-Labrador, Víctor Baselga-Moreno, Miguel Tortajada-Girbés, Juan Mollar-Maseres, Mario Carballido-Fernández, Germán Schwarz-Chavarri, Joan Puig-Barberà, Javier Díez-Domingo, on behalf of the Valencia Hospital Network for the Study of Influenza and Respiratory Viruses Disease

**Affiliations:** 1 Fundación para el Fomento de la Investigación Sanitaria y Biomédica de la Comunitat Valenciana (FISABIO-Public Health), Valencia, Spain; 2 Consorcio de Investigación Biomédica de Epidemiología y Salud Pública (CIBERESP), Instituto de Salud Carlos III, Madrid, Spain; 3 Hospital Doctor Peset, Valencia, Spain; 4 Hospital Universitario y Politécnico La Fe, Valencia, Spain; 5 Hospital General Universitario de Castellón, Castellón, Spain; 6 Universidad CEU Cardenal Herrera, Castellón, Spain; 7 Hospital General de Alicante, Alicante, Spain; 8 Centro de Salud Pública de Castellón, Castellón, Spain; 9 The Network members are acknowledged at the end of the article

**Keywords:** influenza virus, surveillance, vaccine, epidemiology, hospitalisations, Spain, viral infections, influenza

## Abstract

**Introduction:**

Influenza immunisation is recommended for elderly people each season. The influenza vaccine effectiveness (IVE) varies annually due to influenza viruses evolving and the vaccine composition.

**Aim:**

To estimate, in inpatients ≥ 60 years old, the 2017/18 trivalent IVE, overall, by vaccine type and by strain. The impact of vaccination in any of the two previous seasons (2016/17 and 2015/16) on current (2017/18) IVE was also explored.

**Methods:**

This was a multicentre prospective observational study within the Valencia Hospital Surveillance Network for the Study of Influenza and Respiratory Viruses Disease (VAHNSI, Spain). The test-negative design was applied taking laboratory-confirmed influenza as outcome and vaccination status as main exposure. Information about potential confounders was obtained from clinical registries and/or by interviewing patients; vaccine information was only ascertained by registries.

**Results:**

Overall, 2017/18 IVE was 9.9% (95% CI: −15.5 to 29.6%), and specifically, 48.3% (95% CI: 13.5% to 69.1%), −29.9% (95% CI: −79.1% to 5.8%) and 25.7% (95% CI: −8.8% to 49.3%) against A(H1N1)pdm09, A(H3N2) and B/Yamagata lineage, respectively. For the adjuvanted and non-adjuvanted vaccines, overall IVE was 10.0% (95% CI: −24.4% to 34.9%) and 7.8% (95% CI: −23.1% to 31.0%) respectively. Prior vaccination significantly protected against influenza B/Yamagata lineage (IVE: 50.2%; 95% CI: 2.3% to 74.6%) in patients not vaccinated in the current season. For those repeatedly vaccinated against influenza A(H1N1)pdm09, IVE was 46.4% (95% CI: 6.8% to 69.2%).

**Conclusion:**

Our data revealed low vaccine effectiveness against influenza in hospitalised patients ≥60 years old in 2017/18. Prior vaccination protected against influenza A(H1N1)pdm09 and B/Yamagata-lineage.

## Introduction

The World Health Organization (WHO) establishes that vaccination is the most effective way to prevent infection and severe outcomes caused by influenza viruses. Influenza vaccination is widely recommended for preventing seasonal influenza [1], especially for the elderly (≥ 65 years old) as they represent around 90% of all influenza-related deaths [2,3].

Influenza vaccines need to be reformulated each season due to the constant evolution of influenza viruses as well as the circulation of different influenza virus types from one season to another [4]. Health authorities decide on the vaccine composition for an upcoming season before the end of the previous season based on the information provided by the WHO Global Influenza Surveillance and Response System [5].

The trivalent influenza vaccine (TIV) for the 2017/18 northern hemisphere season included influenza A/Michigan/45/2015(H1N1)-like, A/HongKong/4801/2014(H3N2)-like and B/Brisbane/60/2008(Victoria-lineage)-like antigens [5] and was offered free of charge for persons aged ≥ 60 years in the Valencia Region of Spain. The vaccine impact on infections with influenza A(H3N2) for this season was expected to be low, as subclades 3C.2a1, 3C.2a2, 3C.2a3 and 3C.2a4 emerged within vaccine virus clade 3C.2a, each subclade with particular mutations compared with the A/HongKong/4801/2014(H3N2) vaccine component [6]. Although different viruses’ distribution patterns were observed among European countries [7], the 2017/18 season was characterised in Europe by the co-circulation of influenza A(H1N1)pdm09, A(H3N2) and B/Yamagata lineage [8,9], the latter not included in the trivalent vaccine.

Interim effectiveness analysis in the 2017/18 season in Europe found a moderate effectiveness of the vaccine to prevent any influenza, including influenza B/Yamagata lineage despite not being included in the vaccine [10,11]. The impact of previous vaccinations on current season IVE has been widely discussed [12,13]. Repeated vaccination has been observed to impair vaccine effectiveness against A(H1N1)pdm09 [14] and against A(H3N2) [15]. Several studies, however, encourage current season vaccination regardless of vaccination history [14,16], arguing that repeated vaccination is protective even in the presence of potential vaccination interference, with similar results in hospitalised and ambulatory patients [17-19]. In hospitalised older adults (≥ 65 years old), repeated vaccination has been reported twice as effective in preventing severe influenza compared with non-severe influenza [19].

Since 2009, the Valencia Hospital Network for the Study of Influenza (VAHNSI) has conducted annually a prospective active-surveillance hospital-based study in the Valencia Region in Spain to explore the epidemiology of influenza viruses and to estimate the influenza vaccine effectiveness (IVE) in hospitalised patients against laboratory-confirmed influenza (LCI) [20-22].

We report here IVE estimates in hospitalised patients ≥ 60 years old against LCI in the 2017/18 influenza season in the Valencia Region in Spain. IVE was estimated for all influenza, by influenza strain and by vaccine type. The impact of prior vaccination was also estimated considering the vaccination history in the two previous influenza seasons.

## Methods

### Study procedures

The prospective active-surveillance observational study was carried out in four hospitals in the Valencia Region: Hospital General de Castellón (Castellón, Spain), Hospital La Fe (Valencia, Spain), Hospital Doctor Peset (Valencia, Spain) and Hospital General de Alicante (Alicante, Spain). Those hospitals provided healthcare to 1,105,570 (22%) inhabitants of the Valencia Region.

Study procedures have been previously described [21]. Briefly, study staff screened consecutive hospitalised patients who had been discharged from the emergency department in order to be further admitted as inpatients. Patients were eligible for the study if they were ≥ 60 years old, admitted in hospital through the emergency department with a diagnosis possibly related to influenza, resident in one of the participating hospitals’ catchment area, not institutionalised and not discharged from a previous hospitalisation episode in the 30 days prior to the current admission. For inclusion in the analysis, patients had to have signed a written informed consent and had to have reported symptoms of influenza-like illness (ILI, defined as per the European Union ILI-case definition [23], as fever or feverishness, malaise, myalgia or headache and shortness of breath, sore throat or cough), which had occurred in the 7 days prior to admission to the emergency department. Individuals were considered immunised if they had received the current season’s influenza vaccine at least 15 days before symptoms onset. Vaccinated patients who were not immunised at the time of onset of symptoms were excluded from the analysis. The analysis was restricted to patients  who had been recruited during the influenza season, defined as the period between the first of two consecutive weeks with two or more influenza cases detected in our hospital network and the previous week to the first of two consecutive weeks with no influenza cases detected in the network. Patients with damaged samples, pending laboratory results or missing data on the laboratory results outcome, main exposure variable (i.e. vaccination status or vaccine type) or covariates included in the model were excluded from the analysis.

### Ethical statement

The Ethics Research Committee of the Dirección General de Salud Pública-Centro Superior de Investigación en Salud Pública (DGSP-CSISP) approved the protocol of the study. All patients signed a written informed consent before their inclusion in the study.

### Vaccine Information System

Information related to influenza vaccination such as administration date of the vaccine, brand, batch and manufacturer was obtained from the Valencia Region Vaccine Information System (VRVIS) for all patients included in the study. VRVIS is a population-based register that records vaccine doses given at public and private healthcare facilities (primary care centres, hospitals, residential facilities in the public sector and any private sector healthcare facility that applies for access). The sensitivity and specificity of VRVIS was estimated to be 90% and 99%, respectively [21,24]. The VRVIS is linked to inpatient and outpatient clinical records and sociodemographic information through a personal identification number.

### Laboratory procedures

Nasopharyngeal and pharyngeal swabs were obtained within the first 48 hours of admission from patients fulfilling the inclusion criteria. Both swabs were combined in one tube of viral transport media (Copan, Italy) and shipped refrigerated to a centralised virology laboratory at FISABIO-Public Health. One third of the viral transport media volume was used for extraction of total nucleic acids using an automated silica-based method (Nuclisens Easy-Mag, BioMérieux, Lyon, France). Extracted nucleic acids were tested for influenza viruses by multiplex real-time reverse transcription-PCR (RT-PCR), following WHO protocols [25] with the qScript XLT One-Step RT-qPCR ToughMix (Quanta BioSciences, Maryland, United States (US)) in a Lightcycler 480II apparatus (Roche Diagnostics, Spain). First, a real-time RT-PCR screening assay was performed to detect and differentiate influenza A and B viruses using different primers and probes for the matrix protein [26]. Thereafter, two different real-time RT-PCR typing assays were performed to determine the viral subtype/lineage of influenza A or B viruses on influenza-positive samples [27,28].

Molecular characterisation of influenza A(H3N2), A(H1N1)pdm09, B/Yamagata or B/Victoria viruses was performed by haemagglutinin (HA) gene sequencing. All isolates from hospitalised cases with sufficient viral load (Ct < 25) were systematically selected and a specific end-point RT-PCR amplification protocol was applied using different HA-specific primer sets for the corresponding virus type and subtype [28]. The amplified fragments (complete HA coding region) were sequenced by the Sanger method with the BigDye Direct Cycle Sequencing Kit in an ABI 3730xl DNA sequencer (Applied Biosystems, Life Technologies, Foster City, California, US) using specific primers to the corresponding virus type and subtype [28] at the Genomics Core of the Servei Central de Suport a la Investigació Experimental (SCSIE) in the University of Valencia, Spain. The obtained sequences were deposited in the Global Initiative on Sharing All Influenza Data (GISAID) database under accession numbers: EPI_ISL_369223, EPI_ISL_369224, EPI_ISL_369225, EPI_ISL_369226, EPI_ISL_369227, EPI_ISL_369228, EPI_ISL_369229, EPI_ISL_369230, EPI_ISL_369231, EPI_ISL_369232, EPI_ISL_369233, EPI_ISL_369234, EPI_ISL_369235, EPI_ISL_369236, EPI_ISL_369237, EPI_ISL_369238, EPI_ISL_369239, EPI_ISL_369240, EPI_ISL_369241, EPI_ISL_369242, EPI_ISL_369243, EPI_ISL_369244, EPI_ISL_369245, EPI_ISL_369246, EPI_ISL_369247, EPI_ISL_369248, EPI_ISL_369249, EPI_ISL_369250, EPI_ISL_369251, EPI_ISL_369252, EPI_ISL_369253, EPI_ISL_369254, EPI_ISL_369255, EPI_ISL_369256, EPI_ISL_369257, EPI_ISL_369258, EPI_ISL_369259, EPI_ISL_369260, EPI_ISL_369261, EPI_ISL_369262, EPI_ISL_369263, EPI_ISL_369264, EPI_ISL_369265, EPI_ISL_369266, EPI_ISL_369267, EPI_ISL_369268, EPI_ISL_369269, EPI_ISL_369270, EPI_ISL_369271, EPI_ISL_369272, EPI_ISL_369273, EPI_ISL_369274, EPI_ISL_369275, EPI_ISL_369276, EPI_ISL_369277, EPI_ISL_369278, EPI_ISL_369279, EPI_ISL_369280, EPI_ISL_369281, EPI_ISL_369447, EPI_ISL_369448, EPI_ISL_369449, EPI_ISL_369450, EPI_ISL_369451, EPI_ISL_369452, EPI_ISL_369453, EPI_ISL_369454, EPI_ISL_369455, EPI_ISL_369456, EPI_ISL_369457, EPI_ISL_369458, EPI_ISL_369459, EPI_ISL_369460, EPI_ISL_369461, EPI_ISL_369462, EPI_ISL_369463, EPI_ISL_369464, EPI_ISL_369465, EPI_ISL_369466, EPI_ISL_369467, EPI_ISL_369468, EPI_ISL_369469, EPI_ISL_369470, EPI_ISL_369471, EPI_ISL_369472, EPI_ISL_369473, EPI_ISL_369474, EPI_ISL_369475, EPI_ISL_369520.

### Genetic analysis of influenza viruses

Genetic characterisation of influenza A(H3N2), A(H1N1)pdm09, B/Yamagata or B/Victoria viruses was performed by comparison of the obtained HA sequences from the clinical isolates with representative and reference HA sequences (Supplement S1) obtained from the GISAID database (www.gisaid.org). An alignment of reference sequences with sample sequences was generated with the Clustal W algorithm integrated in the BioEdit software version 7.2.5 (http://www.mbio.ncsu.edu/bioedit/bioedit.html). Phylogenetic trees were inferred using maximum-likelihood methods and the best-fitting nt substitution model with the online PhyML platform (http://www.atcg-montpellier.fr/phyml). Branch reliability was evaluated by approximate likelihood-ratio tests [29].

### Statistical analysis

Differences between LCI and non-LCI hospitalised patients were assessed performing a Chi-squared test or a Fisher exact test as appropriate. We used the same tests when comparing vaccinated and unvaccinated individuals. All probabilities were two-tailed and p values under 0.05 were considered statistically significant.

The test-negative design, a variation of the case–control study, was used to estimate IVE [30,31]. According to this approach, patients were enrolled, in our case in hospitals, based on an established case definition. Cases were LCI admitted patients and controls were non-LCI admitted patients. The adjusted odds ratio (aOR) was estimated using a mixed effects logistic regression model including potential confounders such as age, sex, number of underlying conditions, obesity status (obese defined as a body mass index, BMI, ≥ 30), previous admission in the last 12 months, number of general practitioner (GP) consultations in the last 3 months, smoking habits, socioeconomic status according to occupation [32], days from onset of symptoms to swabbing and hospital as fixed effects, and epidemiological week at admission as random effect. IVE was calculated as (1 − aOR) × 100%, comparing the odds of vaccination among LCI cases and non-LCI cases. Analyses were repeated by strain (A(H3N2), A(H1N1)pdm09, B/Yamagata lineage), by vaccine type (adjuvanted or non-adjuvanted) and according to current and prior two seasons influenza vaccination taking no vaccination in any of the three considered seasons as the reference category.

All statistical analyses were carried out in Stata version 14 (StataCorp, College Station, Texas).

## Results

### Study participants and period

**Figure 1:**
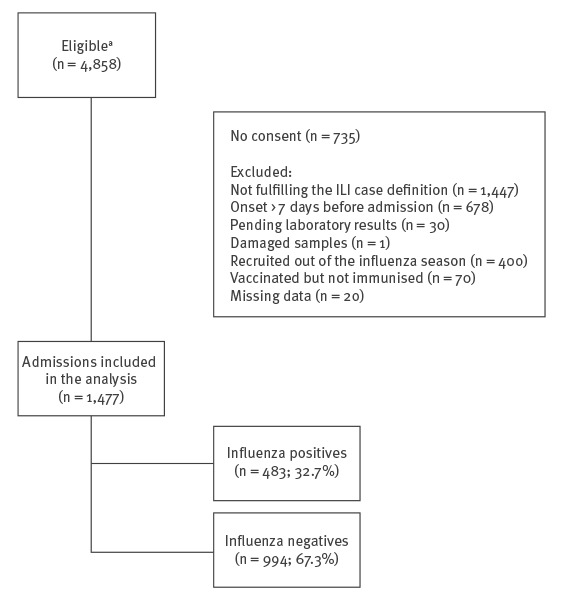
Selection process and influenza status of hospitalised patients ≥ 60 years old for the influenza vaccine effectiveness study, Valencia Hospital Network for the Study of Influenza (VAHNSI), Spain, 2017/18 influenza season (n = 4,858 eligible patients)

**Figure 2:**
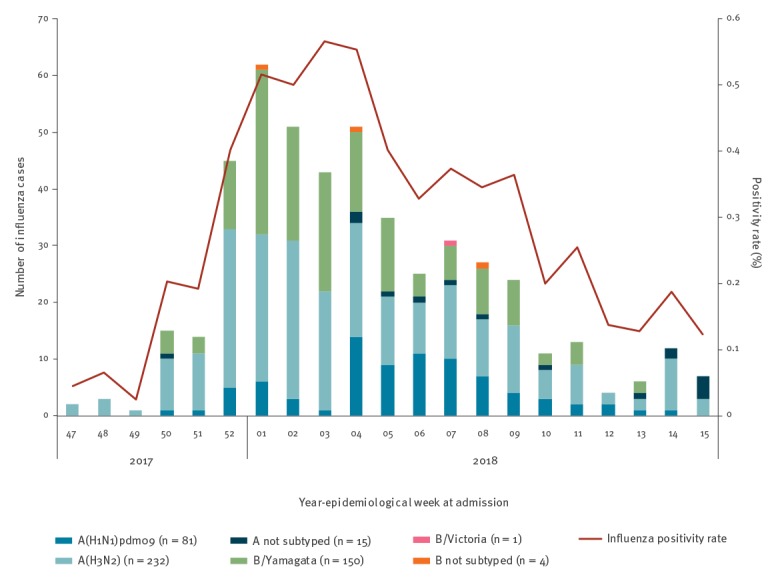
Admissions with laboratory-confirmed influenza in patients ≥ 60 years old, with influenza positivity percentages shown by epidemiological week, Valencia Hospital Network for the Study of Influenza (VAHNSI), Spain, 2017/18 influenza season (n = 483 patients)

After excluding from eligible persons, individuals who did not fulfil the inclusion criteria for the study, the analysis comprised a total of 1,477 hospital admissions among patients ≥ 60 years old (Figure 1). The VAHNSI 2017/18 influenza period was from week 47 of 2017 to week 15 of 2018 (Figure 2). The first LCI and last LCI cases were admitted on 20 November 2017 and the 11 April 2018, respectively.

ILI: influenza-like illness.

^a^ Individuals were eligible if they were patients ≥ 60 years old, admitted in hospital through the emergency department with a diagnosis possibly related to influenza, resident in one of the participating hospitals’ catchment area, not institutionalised and not discharged from a previous hospitalisation episode in the 30 days prior to the current admission.

Our data revealed that the 2017/18 influenza season in the Valencia Region in Spain was characterised by the co-circulation of influenza A(H1N1)pdm09, A(H3N2) and B/Yamagata lineage viruses. The epidemic waves of influenza A(H3N2) and B/Yamagata lineage viruses were situated at the beginning of the season and the wave of influenza A(H1N1)pdm09 viruses in the second half of the influenza season (Figure 2).

### Influenza positives vs influenza negatives

A total of 483 hospitalised patients (32.7%) were LCI. Of these, 81 were infected with influenza A(H1N1)pdm09 (16.8%), 232 A(H3N2) (48.0%), 150 B/Yamagata lineage (31.1%) virus strains and one was infected with an influenza B/Victoria lineage virus (0.2%). Fifteen (3.1%) influenza A and four (0.8%) influenza B infected patients remained not subtyped because of low viral loads (Table 1).

**Table 1:** Characteristics of patients ≥ 60 years old admitted to hospital and included in the influenza vaccine effectiveness study, Valencia Hospital Network for the Study of Influenza (VAHNSI), Spain, 2017/18 influenza season (n = 1,477 patients)

Characteristics	Influenza positive		Influenza negative		p value^c^	Number vaccinated in 2017/18	Total	%^d^	p value
	n	%^a^	n	%^b^					
**Overall (n** **=** **1,477)**	483	32.7	994	67.3	NA	759	1,477	51.4	NA
**Age in years**									
60–69	88	18.2	203	20.4	0.218	99	291	34.0	**< 0.001**
70–79	166	34.4	289	29.1		244	455	53.6	
80–89	177	36.6	384	38.6		327	561	58.3	
≥ 90	52	10.8	118	11.9		89	170	52.4	
**Sex**									
Male	234	48.4	526	52.9	0.107	420	760	55.3	**0.002**
Female	249	51.6	468	47.1		339	717	47.3	
**Underlying conditions (number)**									
None	29	6.0	76	7.6	0.181	35	105	33.3	**< 0.001**
One	122	25.3	214	21.5		154	336	45.8	
Two or more	332	68.7	704	70.8		570	1,036	55.0	
**Admission in the last 12** **months**									
Yes	140	29.0	346	34.8	**0.025**	485	991	48.9	**0.007**
No	343	71.0	648	65.2		274	486	56.4	
**GP visits in the last 3** **months**									
None	145	30.0	314	31.6	0.814	223	459	48.6	0.126
One	56	11.6	116	11.7		82	172	47.7	
Two or more	282	58.4	564	56.7		454	846	53.7	
**Smoking habits**									
Never	256	53.0	427	43.0	**< 0.001**	350	683	51.2	**< 0.001**
Ex-smoker	158	32.7	425	42.8		336	583	57.6	
Current smoker	69	14.3	142	14.3		73	211	34.6	
**Socioeconomic status^e^**									
Professional	57	11.8	119	12.0	0.953	88	176	50.0	0.527
Skilled	43	8.9	93	9.4		76	136	55.9	
Unskilled	383	79.3	782	78.7		595	1,165	51.1	
**Obesity** ^f^									
No	356	73.7	733	73.7	0.988	556	1,089	51.1	0.669
Yes	127	26.3	261	26.3		203	388	52.3	
**Days from onset to swab**									
0–2	74	15.3	175	17.6	0.310	131	249	52.6	0.153
3–4	212	43.9	387	38.9		300	599	50.1	
5–7	157	32.5	348	35.0		274	505	54.3	
> 7	40	8.3	84	8.5		54	124	43.5	
**Current and prior vaccination**									
Vaccinated 2017/18	242	50.1	517	52.0	0.491	NA	NA	NA	NA
Vaccinated 2016/17	241	49.9	528	53.1	0.245	663	769	86.2	**< 0.001**
Vaccinated 2015/16	254	52.6	544	54.7	0.439	651	798	81.6	**< 0.001**
**Influenza test results^g^**									
Negative	0	0.0	994	100.0	NA	517	994	52.0	0.491
A(H1N1)pdm09	81	16.8	0	0.0	NA	30	81	37.0	**0.008**
A(H3N2)	232	48.0	0	0.0	NA	132	232	56.9	0.067
B/Yamagata lineage	150	31.1	0	0.0	NA	69	150	46.0	0.405
B/Victoria lineage	1	0.2	0	0.0	NA	1	1	0.0	0.272

GP: general practitioner.

^a^ Except for the ‘Overall’ category line of the Table, where the percentages are calculated relative to the total number of patients included in the analysis (i.e. 1,477), the rest of the percentages presented in this column are based on the total of patients testing positive for influenza (i.e. 483).

^b^ Except for the ‘Overall’ category line of the Table, where the percentages are calculated relative to the total number of patients included in the analysis (i.e. 1,477), the rest of the percentages presented in this column are based on the total of patients testing negative for influenza (i.e. 994).

^c^ These p values identify whether there is a dependence between the laboratory-confirmed influenza variable and the characteristics on the left, e.g. age. P values < 0.05 indicate that influenza cases and influenza controls were not equally distributed according to the explored characteristics on the left.

^d^ Percentages in this column are based on the numbers and totals displayed in the two previous columns.

^e^ Socioeconomic status: ‘professional’ includes professionals, managers, medium or superior technicians, small entrepreneurs, middle managers, supervisors; ‘skilled’ includes skilled manual workers; ‘unskilled’ includes semi-skilled and unskilled manual workers.

^f^ Obesity was defined as a body mass index (BMI) ≥ 30.

^g^ Fifteen influenza A and four influenza B samples were not subtyped because of low viral loads.

Bold font is used to highlight p values indicating statistical significance.

Hospitalisation during the preceding year was more common among non-LCI than LCI hospitalised patients (34.8 vs 29.0%). In terms of smoking habits, most of the LCI hospitalised patients (53.0%) never smoked vs 43.0% of non-LCI admissions. (Table 1).

### Vaccinated vs unvaccinated individuals

Overall, 1,240 (84.0%) individuals had a vaccination record for any type of vaccine (influenza or other) in VRVIS. Of the 237 (16.0%) individuals not in the registry, 230 (97.0%) reported not being vaccinated. The 237 patients not included in the registry were considered as not vaccinated. Overall, 759 (51.4%) and 718 (48.6%) admissions were in vaccinated and unvaccinated patients with the 2017/18 seasonal influenza vaccine, respectively. Vaccination coverage increased significantly with age and with the number of underlying conditions. Most of the vaccinated individuals were men (55.3%; 420/759). Never-smokers or ex-smokers were more often vaccinated than current smokers (51.2% and 57.6% vs 34.6%; p < 0.001). Most of the patients vaccinated in the current season were also vaccinated in the two previous seasons, with 663 of them (87.3%) also vaccinated in 2016/17 and 651 (85.8%) also vaccinated in 2015/16 (Table 1). Among vaccinees, 339 (44.7%) received the adjuvanted vaccine and 411 (54.1%) the non-adjuvanted vaccine. Vaccine type was unknown for nine (1.2%) vaccinated individuals (data not shown).

### Viral genetic analysis

The detailed genetic characterisation, mutational pattern and concordance of the different genetic groups with the vaccine and reference strains was performed using the complete HA coding sequence. Influenza A(H3N2) viruses in circulation in Europe at the time of sampling corresponded to the genetic clades 3C.3a and 3C.2a, with clade 3C.2a viruses predominating, but with the HA gene sequences characterised by a quite divergent genetic composition. Among clade 3C.2a, new subclades and subgroups have emerged (i.e. subclades 3C.2a1, 3C.2a2, 3C.2a3 and 3C.2a4) [33].

All the 33 A(H3N2) isolates sequenced in this study corresponded to either subclades 3C.2a1 (A/Singapore/INFIMH-16–0019/2016-like) (n = 11) or 3C.2a2 (A/Nantes/1441/2017-like) (n = 22) viruses, different to the A/HongKong/4801/2014 (clade 3C.2a) vaccine virus, and including changes in antigenic sites and in glycosylation patterns. All 11 3C.2a1 viruses could be further classified as subgroup 3C.2a1b (A/Singapore/INFIMH-16–0019/2016-like), with in HA1 a N121K (site D) mutation and in HA2, I406V and G484E, when compared with the A/HongKong/4801/2014 vaccine virus. Additional mutational patterns further characterised three types of isolates: (i) E62G + Q80K + K92R (site D) + N122D + T135K (site A, loss of glycosylation) + R142G (site A)  in HA1 and S432T in HA2 (n = 3, including two from vaccinees); (ii) E62G + K92R (site D) + N122D + T135K (site A, loss of glycosylation) + R142G (site A) in HA1 and S432T in HA2 (n = 1, from a vaccinee); and (iii) E62G + K92R (site D) + T128A + T135K (site A, loss of glycosylation) + R142G (site A) in HA1 (n = 7, four from vaccinees). In the 22 subclade 3C.2a2 viruses, two main patterns were observed when compared with the A/HongKong/4801/2014 vaccine virus: (i) R142K (site A) + R261Q alone (n = 1, from vaccinee); and (ii) T131K + R142K (site A) + R261Q (site E) as a common pattern (n = 21, 13 from vaccinees). The latter viruses could be further differentiated in those without additional mutations (n = 13, nine from vaccinees); and those with additional mutations Y94K (site E) (n = 1, from a non-vaccinee); S144R (site A) (n = 5, two from vaccinees); or S144R (site A) + S265G (n = 2, both from vaccinees).

All nine A(H1N1)pdm09 viruses sequenced fell into clade 6B.1 within the A/Paris/1447/2017 subgroup, differentiated from the A/Michigan/45/2015 vaccine virus by mutations S74R (site Cb), S164T (site Sa, linked to change in glycosylation) and I295V; with (n = 4, one from vaccinee) or without (n = 5, two from vaccinees) the additional mutation T120A.

We detected only one case (a vaccinated individual) infected with an influenza B/Victoria lineage virus. The viral sequence corresponded to a clade 1A sequence with I117V + N129D and a two amino acid deletion (162–163) in the HA gene, both characteristic for a new genetic group B/Norway/2409/2017-like.

The 25 B/Yamagata lineage sequenced viruses all corresponded to clade 3, B/Phuket/3073/2013-like, but differed by mutations L172Q + M251V, with few isolates (n = 7) carrying additional mutations H85Y, E141R, V176I, V160I or K335N.

### Vaccine effectiveness in hospitalised patients

Assessed IVE in patients ≥ 60 years old admitted to hospital was 9.9% (95% CI: −15.5% to 29.6%), with no difference between the adjuvanted (IVE: 10.0%; 95% CI: −24.4% to 34.9%) and the non-adjuvanted trivalent vaccine (IVE: 7.8%; 95% CI: −23.1% to 31.0%) (p = 0.1826 for homogeneity of unadjusted odds; data not shown) (Table 2). The impact of previous vaccinations was not significant when considering effect on IVE (Table 3).

**Table 2:** Influenza vaccine effectiveness by vaccine type, regardless of vaccination history in patients ≥ 60 years old admitted to hospital, Valencia Hospital Network for the Study of Influenza (VAHNSI), Spain, 2017/18 influenza season (n **=** 1,477 patients)

Types, subtypes or lineage of influenza	N	Cases^a^	Vaccinated cases			Controls^b^	Vaccinated controls			Overall IVE		Overall IVE (adjuvanted vaccine)		Overall IVE (non-adjuvanted vaccine)	
			Adjuvanted vaccine	Non-adjuvanted vaccine	Total		Adjuvanted vaccine	Non-adjuvanted vaccine	Total	IVE	95% CI	IVE	95% CI	IVE	95% CI
**All influenza^c^**	**1,477**	**483**	**100**	**140**	**240**	**994**	**239**	**271**	**510**	**9.86**	**−15.47 to 29.63**	**9.97**	**−24.43 to 34.86**	**7.82**	**−23.15 to 31.00**
A(H1N1)pdm09^d^	684	77	12	16	28	607	137	189	326	48.33	13.51 to 69.13	34.38	−34.58 to 68.00	54.12	15.32 to 75.14
A(H3N2)^e^	1,226	232	54	77	131	994	239	271	510	−29.88	−79.09 to 5.81	−23.93	−87.94 to 18.28	−37.03	−98.15 to 5.24
B/Yamagata^f^	916	150	29	39	68	766	180	228	408	25.75	−8.83 to 49.35	30.09	−15.94 to 57.85	21.10	−24.43 to 49.97

CI: confidence interval; GP: general practitioner; IVE: influenza vaccine effectiveness; LCI: laboratory-confirmed influenza.

^a^ Cases in this column were individuals included in the study with LCI who were admitted to hospital during the period when influenza viruses of the type or subtype in question circulated. For example, cases of all influenza comprised all LCI individuals admitted during the 2017/18 influenza season, while cases of influenza A(H1N1)pdm09, A(H3N2) and B/Yamagata lineage only included patients admitted during times when influenza A(H1N1)pdm09, A(H3N2) and B/Yamagata lineage viruses respectively circulated. For each virus type, the time of circulation was estimated as the period between the first of at least two consecutive weeks with two or more cases of this type and the previous week of the first of two consecutive weeks with no cases of this type.

^b^ Controls in this column were individuals included in the study who tested negative for influenza in the laboratory and who were admitted to hospital during the time that viruses of the influenza type in question circulated. The time of circulation was estimated as described in the above footnote a.

^c^ Adjusted by age, number of chronic conditions, sex, socioeconomic class (occupation), admission in the last 12 months, number of GP visits in the last 3 months, smoking habits, obesity status, days between symptoms onset and swab, hospital and epidemiological week at admission. Nine individuals vaccinated with a vaccine different from the ones under study were excluded from the IVE estimations by vaccine type (seven controls and two cases).

^d^ Adjusted by age, sex and epidemiological week at admission. Four individuals vaccinated with a vaccine different from the ones under study were excluded from the IVE estimations by vaccine type; all four were controls.

^e^ Adjusted by age, number of chronic conditions, sex, socioeconomic status (occupation), admission in the last 12 months, number of GP visits in the last 3 months, smoking habits, obesity status, days between symptoms onset and swab, hospital and epidemiological week at admission. Eight individuals vaccinated with a vaccine different from the ones under study were excluded from the IVE estimations by vaccine type (seven controls and one case).

^f^ Adjusted by age, number of chronic conditions, sex, smoking habits and epidemiological week at admission. Seven individuals vaccinated with a vaccine different from the ones under study were excluded from the IVE estimations by vaccine type (six controls and one case).

**Table 3:** Influenza vaccine effectiveness, considering vaccination history in the current and the two previous seasons in patients ≥ 60 years old admitted to hospital, Valencia Hospital Network for the Study of Influenza (VAHNSI), Valencia, Spain, 2017/18 influenza season (n **=** 1,477 patients)

Types, subtypes or lineage of influenza	Vaccinated in either 2015/16 or 2016/17^a^	Vaccinated in 2017/18	IVE^b^	95% CI
All influenza^c^	No	Yes	30.16	−34.21 to 63.65
	Yes	Yes	14.20	−12.79 to 34.73
	Yes	No	22.98	−16.64 to 49.14
A(H1N1)pdm09^d^	No	Yes	80.11	−53.74 to 97.43
	Yes	Yes	46.41	6.78 to 69.20
	Yes	No	10.60	−94.00 to 58.80
A(H3N2)^e^	No	Yes	−1.47	−142.01 to 57.46
	Yes	Yes	−35.26	−93.67 to 5.53
	Yes	No	−7.52	−84.80 to 37.44
B/Yamagata^f^	No	Yes	4.48	−142.60 to 62.39
	Yes	Yes	39.61	8.13 to 60.31
	Yes	No	50.18	2.34 to 74.59

CI: confidence interval; GP: general practitioner; IVE: influenza vaccine effectiveness.

^a^ The 2015/16 influenza vaccine comprised an A/California/7/2009 (H1N1)pdm09-like virus, an A/Switzerland/9715293/2013 (H3N2)-like virus and a B/Phuket/3073/2013-like virus (Yamagata lineage). The 2016/17 influenza vaccine comprised an A/California/7/2009 (H1N1)pdm09-like virus, an A/Hong Kong/4801/2014 (H3N2)-like virus and a B/Brisbane/60/2008-like virus (Victoria lineage).

^b^ Taking those individuals not vaccinated in any of the three considered seasons as reference category.

^c^ Adjusted by age, number of chronic conditions, sex, socioeconomic status (occupation), admission in the last 12 months, number of GP visits in the last 3 months, smoking habits, obesity status, days between symptoms onset and swab, hospital and epidemiological week at admission.

^d^ Adjusted by age, sex and epidemiological week at admission.

^e^ Adjusted by age, number of chronic conditions, sex, socioeconomic status (occupation), admission in the last 12 months, number of GP visits in the last 3 months, smoking habits, obesity status, days between symptoms onset and swab, hospital and epidemiological week at admission.

^f^ Adjusted by age, number of chronic conditions, sex, smoking habits and epidemiological week at admission.

### Vaccine effectiveness against A(H1N1)pdm09

IVE assessed in hospitalised patients ≥ 60 years old against A(H1N1)pdm09 was 48.3% (95% CI: 13.5% to 69.1%) (Table 2). IVE was 34.4% (95% CI: −34.6% to 68.0%) for the adjuvanted vaccine and 54.1% (95% CI: 15.3% to 75.1%) for the non-adjuvanted vaccine (p = 0.9319 for homogeneity of unadjusted odds; data not shown) (Table 2). When considering vaccination history and taking those individuals not vaccinated in any of the three considered seasons as reference category, we observed an IVE of 46.4% (95% CI: 6.8% to 69.2%) in those individuals vaccinated in the current and in any of the two previous seasons. No statistically significant differences were found for those vaccinated only in the current season and for those vaccinated in any of the two previous seasons but not in the current one (Table 3).

### Vaccine effectiveness against A(H3N2)

IVE assessed in hospitalised patients ≥ 60 years old against A(H3N2) was −29.9% (95% CI: −79.1% to 5.8%) (Table 2). IVE was −23.9% (95% CI: −87.9% to 18.3%) for the adjuvanted vaccine and −37.0% (95% CI: −98.1% to 5.2%) for the non-adjuvanted vaccine (p = 0.2480 for homogeneity of unadjusted odds; data not shown) (Table 2). The impact of previous vaccinations was not significant when considering effect on IVE (Table 3).

### Vaccine effectiveness against B/Yamagata-lineage

IVE assessed in hospitalised patients ≥ 60 years old against B/Yamagata lineage was 25.7% (95% CI: −8.8% to 49.3%) (Table 2). IVE was 30.1% (95% CI: −15.9% to 57.8%) for the adjuvanted vaccine and 21.1% (95% CI: −24.4% to 50.0%) for the non-adjuvanted vaccine (p = 0.8212 for homogeneity of unadjusted odds; data not shown) (Table 2). When considering vaccination history and taking those individuals not vaccinated in any of the three considered seasons, we observed an IVE of 39.6% (95% CI: 8.1% to 60.3%) in those individuals vaccinated in the current and in any of the two previous seasons and an IVE of 50.2% (95% CI: 2.3% to 74.6%) for those not vaccinated in the current season but in any of the two previous ones. No effectiveness (IVE: 4.5; 95% CI: −142.6% to 62.4%) was observed for those vaccinated only in the current season (Table 3).

## Discussion

During the 2017/18 season we observed non-significant PCR-confirmed influenza vaccine effectiveness in hospitalised patients ≥ 60 years of age. There was nevertheless significant protection against influenza A(H1N1)pdm09, a subtype that circulated at low levels in Spain. In the country, the influenza season started earlier than in previous years and persisted for 21 weeks, up to 5 weeks longer than in the 2016/17 season [34,35], therefore having a higher social impact.

The 2017/18 influenza season in the northern hemisphere was characterised by co-circulation of different viruses, including A(H1N1)pdm09, A(H3N2) and B. Between 88 to 94% of the influenza B viruses corresponded to the Yamagata lineage [10,11,36], mainly B/Phuket/3073/2013(Yamagata-lineage)-like strain [10].

Most of the influenza A(H3N2) isolates characterised in the current study corresponded to subclades 3C.2a1 or 3C.2a2, with several mutations, compared with the clade 3C2a A/HongKong/4801/2014 vaccine strain, including several antigenic sites and changes in glycosylation patterns. In the northern hemisphere, 63–71% of A(H3N2) isolated viruses belonged to the A/HongKong/4801/2014-like strain, related to past season’s A/Bolzano/7/2016 (3C.2a clade), which only represented 26% of the positives in the Spanish sentinel influenza network [37]. In Canada 93% of the characterised A(H3N2) viruses were clade 3C.2a. Antisera raised against the egg-propagated vaccine virus A/HongKong/4801/2014 recognised a small minority of circulating viruses in 3C.2a subclades this season [38,39], suggesting a potential antigenic mismatch that could explain in part the poor IVE for the A(H3N2) component.

The A(H1N1)pdm09 viruses analysed were clade 6B.1 with different mutations, but these HA variants have been reported to be antigenically similar to the A/Michigan/45/2015 vaccine-virus [40]. There was a moderate vaccine effectiveness against this virus, indicating that the antigenic mismatch between the circulating virus and the vaccine component was probably low. However, non-significant IVE was found in our study against influenza A(H1N1)pdm09 in those vaccinated in the current season but not in any of the two previous seasons. Repeated vaccination against this influenza strain improved the IVE estimate although the strain included in the vaccine of the two previous seasons was A/California/7/2009 (H1N1)pdm09-like virus. Caution should be taken because of small numbers when dividing individuals into groups. Our study focused on patients 60 years old or over and it is very common that those who were vaccinated in the current season were also vaccinated in the previous ones, resulting in a small number in the vaccinated only in the current season category.

Most of the influenza B viruses isolated in our study (150/155) corresponded to the Yamagata lineage, and the 25 sequenced isolates all belonged to clade 3, similar to the B/Phuket/3073/2013 vaccine strain. Although they showed additional substitutions (i.e. L172Q + M251V), these variant viruses seem well recognised by antiserum raised against B/Phuket/3073/2013 vaccine virus, which belongs to the Yamagata lineage [38,39]. The B/Yamagata lineage was however not included in the 2017/18 trivalent vaccine and our study found low, non-significant trivalent IVE against influenza B/Yamagata-lineage. Mid-season reports in Europe [11] found decreasing protection of the vaccine against this mismatched lineage with age. In cases of influenza B lineage mismatch between vaccine and circulating strains, a certain level of protection is expected as a result of residual effect of prior years’ vaccination with the circulating lineage, and some degree of cross-reactivity [41]. These reasons could explain the advantage of the repeated vaccination against influenza B/Yamagata lineage we found in our study. Influenza B lineages are antigenically different and virus neutralising antibodies poorly cross-react between lineages [42]. However, studies in ferrets showed evidence of viral interference and cross-reactive immunity, with animals infected with one B lineage showing some degree of protection against subsequent challenge with either B lineage [43]. This effect could be due to CD8 + cytotoxic T-cells directed to one B lineage cross-reacting with antigens of the other lineage, even in absence of neutralising antibodies [42].

Concerning all influenza, due to the divergence between the circulating viruses and the trivalent vaccine content, a low vaccine effectiveness was expected [6,44]. In fact, IVE against all influenza was low and none of the two vaccines (adjuvanted or non-adjuvanted) offered significant protection. Mid-season interim reports, mainly describing outpatients surveillance systems in Europe [10,11] already showed moderate protection, especially to A(H1N1)pdm09 and B infections and in younger populations. Although a moderate protection was reached against influenza A(H1N1)pdm09 in our study, the low circulation of this subtype in Spain prevented a large health impact of the vaccine. IVE against influenza A(H3N2) was null overall and by vaccine type. This finding is consistent with results obtained by other studies [10,11,36] in any age group.

Some published studies suggested that IVE is age dependent, with higher effectiveness in younger age groups and lower in the elderly, the groups where vaccination is mostly recommended [45,46]. There are differences among studies that make comparisons difficult, especially the level of care where the cases are collected. In general, IVE studied in hospitalised patients tends to be lower than IVE based on data from surveillance systems. The reason is unclear, but potential biases may be present in either study designs, primary care or hospital settings [47]. When assessing IVE, an ideal active comparator has a similar indication to the treatment or intervention of interest and is administered to a population with a similar distribution of measured and unmeasured patient characteristics [48]. This is sometimes difficult to assess in observational studies and may remain as a residual bias, but may have a lower impact in hospitalised, more fragile patients.

Our study has the typical limitations of an observational study. The absence of statistical significance and wide confidence intervals are common in studies with moderate to low IVE, low vaccine coverage and small sample size [49]. We restricted our analysis to periods with influenza circulation and we only considered patients fulfilling the ECDC ILI-case definition and an onset of symptoms in the 7 days prior to admission to control the heterogeneity in the study due to case ascertainment. Vaccination status was ascertained by registries, influenza was confirmed with a sensitive RT-PCR assay and only patients swabbed within 48 h of admission in hospital were included to avoid misclassification bias.

Our data support the importance of taking into account virological data and influenza vaccine effectiveness results to make better decisions during the challenging task of the seasonal vaccine composition choice. We also add valuable information regarding the controversial issue about the impact of previous vaccinations and influenza vaccine effectiveness for the different commercialised vaccines in the Valencia Region.

## Supplementary Data

86000 Supplementary Material
