# Supplementary material for: Influenza vaccine effectiveness against laboratory-confirmed influenza in hospitalised adults aged 60 years or older, Valencia Region, Spain, 2017/18 influenza season
Source: Euro Surveill. 2019 Aug 1;24(31):1800461. doi: 10.2807/1560-7917.ES.2019.24.31.1800461 (PMC6685101; doi:10.2807/1560-7917.ES.2019.24.31.1800461)
Supplement: Supplementary Material [file 1800461_DIEZ-DOMINGO_influenza_Supplement.pdf]

Supplement S1

This supplementary material is hosted by Eurosurveillance as supporting information alongside the article "Influenza vaccine effectiveness against laboratory-confirmed influenza in hospitalised adults aged 60years or older, Valencia Region, Spain, 2017/18 influenza season" on behalf of the authors who remain responsible for the accuracy and appropriateness of the content. The same standards for ethics, copyright, attributions and permissions as for the article apply. Eurosurveillance is not responsible for the maintenance of any links or email addresses provided therein.

We gratefully acknowledge the authors, originating and submitting laboratories of the sequences from GISAID's EpiFlu™ Database (1) on which this research is based. The list is detailed below. All submitters of data may be contacted directly via the GISAID website www.gisaid.org

Isolate ID	Type	Segment	Collection date	Isolate Name	Originating Laboratory	Submitting Laboratory	Authors
EPI123461	A(H1N1pdm09)	HA	2012-05-21	A/Hong Kong/5659/2012	Hong Kong Department of Health	Hong Kong Department of Health	Mak, Gannon
EPI253573	A(H1N1pdm09)	HA	2017-03-08	A/Michigan/45/2015 (16/354)	National Institute for Biological Standards and Control (NIBSC)		Nicolson, Carolyn
EPI290613	A(H1N1pdm09)	HA	2017-12-04	A/Yunnan-Dali/SWL1406/2017	WHO Chinese National Influenza Center	WHO Chinese National Influenza Center	Zeng, Xiaoxu
EPI291648	A(H1N1pdm09)	HA	2017-11-11	A/Paris/1516/2017	Institut Pasteur	Crick Worldwide Influenza Centre	Gregory, Vicki
EPI291668	A(H1N1pdm09)	HA	2017-10-20	A/Paris/1447/2017	Institut Pasteur	Crick Worldwide Influenza Centre	Gregory, Vicki
EPI291672	A(H1N1pdm09)	HA	2017-10-12	A/Qatar/10-VI-17-0044441/2017	Supreme Health Council	Crick Worldwide Influenza Centre	Gregory, Vicki
EPI292823	A(H1N1pdm09)	HA	2017-10-26	A/Andalucia/2280/2017	Instituto de Salud Carlos III	Crick Worldwide Influenza Centre	Gregory, Vicki
EPI292826	A(H1N1pdm09)	HA	2017-11-13	A/Clermont-Ferrand/2061/2017	CNR Influenza France Sud	Crick Worldwide Influenza Centre	Gregory, Vicki
EPI292831	A(H1N1pdm09)	HA	2017-11-17	A/Ghana/3512/2017	University of Ghana	Crick Worldwide Influenza Centre	Gregory, Vicki
EPI294088	A(H1N1pdm09)	HA	2017-12-04	A/Bayern/95/2017	Robert Koch Institute Nationales Referenzzentrum f³r Influenza	Crick Worldwide Influenza Centre	Ermetal, Burcu
EPI294090	A(H1N1pdm09)	HA	2017-12-20	A/Galicia/2443/2017	Instituto de Salud Carlos III	Crick Worldwide Influenza Centre	Ermetal, Burcu
EPI294092	A(H1N1pdm09)	HA	2017-12-05	A/Ghana/14/2017	University of Ghana	Crick Worldwide Influenza Centre	Ermetal, Burcu
EPI294093	A(H1N1pdm09)	HA	2017-11-29	A/Ghana/3674/2017	University of Ghana	Crick Worldwide Influenza Centre	Ermetal, Burcu
EPI294095	A(H1N1pdm09)	HA	2017-12-10	A/Hong Kong/4979/2017	Government Virus Unit	Crick Worldwide Influenza Centre	Ermetal, Burcu
EPI294096	A(H1N1pdm09)	HA	2017-12-13	A/Hong Kong/4981/2017	Government Virus Unit	Crick Worldwide Influenza Centre	Ermetal, Burcu
EPI294100	A(H1N1pdm09)	HA	2017-12-18	A/Hong Kong/4990/2017	Government Virus Unit	Crick Worldwide Influenza Centre	Ermetal, Burcu
EPI294101	A(H1N1pdm09)	HA	2017-12-18	A/Israel/SK428/2017	Central Virology Laboratory Israel (NIC)	Crick Worldwide Influenza Centre	Ermetal, Burcu
EPI294106	A(H1N1pdm09)	HA	2017-12-13	A/Navarra/2488/2017	Instituto de Salud Carlos III	Crick Worldwide Influenza Centre	Ermetal, Burcu
EPI294109	A(H1N1pdm09)	HA	2017-12-07	A/Norway/3787/2017	WHO National Influenza Centre	Crick Worldwide Influenza Centre	Ermetal, Burcu
EPI294111	A(H1N1pdm09)	HA	2017-12-21	A/Saarland/25/2017	Robert Koch Institute Nationales Referenzzentrum f³r Influenza	Crick Worldwide Influenza Centre	Ermetal, Burcu
EPI294119	A(H1N1pdm09)	HA	2017-12-21	A/Switzerland/2656/2017	Hopital Cantonal Universitaire de Geneves	Crick Worldwide Influenza Centre	Ermetal, Burcu
EPI294121	A(H1N1pdm09)	HA	2017-12-20	A/Switzerland/3330/2017	Hopital Cantonal Universitaire de Geneves	Crick Worldwide Influenza Centre	Ermetal, Burcu
EPI295539	A(H1N1pdm09)	HA	2017-12-30	A/Giresun/612/2017	Refik Saydam National Public Health Agency	Ministry of Health Turkey	Bayrakdar, Fatma
EPI295550	A(H1N1pdm09)	HA	2017-12-30	A/Amasya/621/2017	Refik Saydam National Public Health Agency	Ministry of Health Turkey	Bayrakdar, Fatma
EPI295730	A(H1N1pdm09)	HA	2017-11-01	A/Oman/7149/2017	Central Public Health Laboratory, Ministry of Health	Crick Worldwide Influenza Centre	Gregory, Vicki
EPI295745	A(H1N1pdm09)	HA	2017-12-27	A/Portugal/SU117/2017	Instituto Nacional de Saude (INSA)	Crick Worldwide Influenza Centre	Gregory, Vicki
EPI296034	A(H1N1pdm09)	HA	2017-12-13	A/Louisiana/68/2017	Louisiana Department of Health and Hospitals	Centers for Disease Control and Prevention	DaSilva, Juliana
EPI297802	A(H1N1pdm09)	HA	2017-12-29	A/slovenia/2794/2017	National Laboratory for Health Environment and Food	National Laboratory for Health Environment and Food	Berginc, Natasa
EPI297833	A(H1N1pdm09)	HA	2018-01-05	A/Slovenia/37/2018	National Laboratory for Health Environment and Food	National Laboratory for Health Environment and Food	Berginc, Natasa
EPI302786	A(H1N1pdm09)	HA	2017-12-13	A/KANAGAWA/IC1705/2017	National Institute of Infectious Diseases (NIID)	National Institute of Infectious Diseases (NIID)	Kuwahara, Tomoko
EPI306070	A(H1N1pdm09)	HA	2017-10-22	A/Hong Kong/4820/2017	Government Virus Unit	Centers for Disease Control and Prevention	DaSilva, Juliana
EPI68771	A(H1N1pdm09)	HA	2009	A/Lviv/N6/2009		Centers for Disease Control and Prevention	Garten, Rebecca
EPI73686	A(H1N1pdm09)	HA	2009	A/Bayern/69/2009	Robert Koch Institute Nationales Referenzzentrum für Influenza	Robert Koch Institute Nationales Referenzzentrum f³r Influenza	Biere, Barbara
EPI90760	A(H1N1pdm09)	HA	2011-02-14	A/St. Petersburg/27/2011	WHO National Influenza Centre Russian Federation	National Institute for Medical Research	Gregory, Vicki
EPI90787	A(H1N1pdm09)	HA	2011-02-28	A/Astrakhan/1/2011	WHO National Influenza Centre Russian Federation	National Institute for Medical Research	Gregory, Vicki
EPI90954	A(H1N1pdm09)	HA	2011-03-14	A/St. Petersburg/100/2011	Russian Academy of Medical Sciences	Centers for Disease Control and Prevention	Garten, Rebecca
EPI297349	A(H3N2)	HA	2017-12-29	A/Portugal/EVA43/2017	Instituto Nacional de Saude (INSA)	Crick Worldwide Influenza Centre	Ermetal, Burcu
EPI168694	A(H3N2)	HA	2014-04-30	A/Hong Kong/5738/2014	National Institute for Medical Research	Centers for Disease Control and Prevention	Garten, Rebecca
EPI223655	A(H3N2)	HA	2016-03-04	A/Oman/2585/2016	Central Public Health Laboratory, Ministry of Health	Crick Worldwide Influenza Centre	Gregory, Vicki
EPI226075	A(H3N2)	HA	2016-04-06	A/Cote D'Ivoire/544/2016	Pasteur Institut of Cote d'Ivoire	Crick Worldwide Influenza Centre	Gregory, Vicki
EPI239775	A(H3N2)	HA	2016-10-22	A/Norway/4293/2016	WHO National Influenza Centre	Crick Worldwide Influenza Centre	Ermetal, Burcu
EPI239785	A(H3N2)	HA	2016-11-03	A/Norway/4436/2016	WHO National Influenza Centre	Crick Worldwide Influenza Centre	Ermetal, Burcu
EPI282636	A(H3N2)	HA	2016-06-13	A/Norway/3806/2016	Crick Worldwide Influenza Centre	WHO Collaborating Centre for Reference and Research on Influenza	Komadina, Naomi
EPI285973	A(H3N2)	HA	2017-11-03	A/Stockholm/44/2017		Public Health Agency of Sweden	Brytting, Maria
EPI285986	A(H3N2)	HA	2017-11-18	A/Trollhattan/50/2017		Public Health Agency of Sweden	Brytting, Maria
EPI288767	A(H3N2)	HA	2017-07-04	A/Egypt/4076/2017	Ministry of Health and Population	Crick Worldwide Influenza Centre	Gregory, Vicki
EPI288786	A(H3N2)	HA	2017-10-11	A/Switzerland/882/2017	Hopital Cantonal Universitaire de Geneves	Crick Worldwide Influenza Centre	Gregory, Vicki
EPI291278	A(H3N2)	HA	2017	A/Greece/4/2017	Crick Worldwide Influenza Centre	WHO Collaborating Centre for Reference and Research on Influenza	Komadina, Naomi
EPI291278	A(H3N2)	HA	2017	A/Greece/4/2017	Crick Worldwide Influenza Centre	WHO Collaborating Centre for Reference and Research on Influenza	Komadina, Naomi
EPI291279	A(H3N2)	HA	2016	A/Norway/4849/2016	Crick Worldwide Influenza Centre	WHO Collaborating Centre for Reference and Research on Influenza	Komadina, Naomi
EPI291280	A(H3N2)	HA	2016	A/Norway/4465/2016	Crick Worldwide Influenza Centre	WHO Collaborating Centre for Reference and Research on Influenza	Komadina, Naomi
EPI291407	A(H3N2)	HA	2017-10-02	A/Norway/3247/2017	WHO National Influenza Centre	Crick Worldwide Influenza Centre	Gregory, Vicki
EPI291409	A(H3N2)	HA	2017-10-17	A/Norway/3283/2017	WHO National Influenza Centre	Crick Worldwide Influenza Centre	Gregory, Vicki
EPI291411	A(H3N2)	HA	2017-10-17	A/Norway/3297/2017	WHO National Influenza Centre	Crick Worldwide Influenza Centre	Gregory, Vicki
EPI291417	A(H3N2)	HA	2017-11-15	A/Iran/93702/2017	Tehran University of Medical Sciences	Crick Worldwide Influenza Centre	Gregory, Vicki
EPI292377	A(H3N2)	HA	2017-10-09	A/Bretagne/1413/2017	Institut Pasteur	Crick Worldwide Influenza Centre	Gregory, Vicki
EPI292379	A(H3N2)	HA	2017-11-27	A/Bretagne/1565/2017	Institut Pasteur	Crick Worldwide Influenza Centre	Gregory, Vicki
EPI292387	A(H3N2)	HA	2017-10-10	A/Nantes/1441/2017	Institut Pasteur	Crick Worldwide Influenza Centre	Gregory, Vicki
EPI292393	A(H3N2)	HA	2017-10-17	A/Qatar/10-VI-17-0045513/2017	Supreme Health Council	Crick Worldwide Influenza Centre	Gregory, Vicki
EPI292398	A(H3N2)	HA	2017-10-14	A/Qatar/16-VI-17-0044871/2017	Supreme Health Council	Crick Worldwide Influenza Centre	Gregory, Vicki
EPI292895	A(H3N2)	HA	2017-10-25	A/England/74380294/2017	Microbiology Services Colindale, Public Health England	Crick Worldwide Influenza Centre	Ermetal, Burcu
EPI292897	A(H3N2)	HA	2017-11-06	A/England/74560298/2017	Microbiology Services Colindale, Public Health England	Crick Worldwide Influenza Centre	Ermetal, Burcu
EPI292902	A(H3N2)	HA	2017-11-17	A/Eskilstuna/4/2017	Public Health Agency of Sweden	Crick Worldwide Influenza Centre	Ermetal, Burcu
EPI292905	A(H3N2)	HA	2017-09-15	A/Ghana/2717/2017	University of Ghana	Crick Worldwide Influenza Centre	Ermetal, Burcu
EPI292906	A(H3N2)	HA	2017-10-10	A/Karlstad/5/2017	Public Health Agency of Sweden	Crick Worldwide Influenza Centre	Ermetal, Burcu
EPI292921	A(H3N2)	HA	2017-10-17	A/Stockholm/43/2017	Public Health Agency of Sweden	Crick Worldwide Influenza Centre	Ermetal, Burcu
EPI293085	A(H3N2)	HA	2017-07-28	A/Hong Kong/4018/2017	Government Virus Unit	Crick Worldwide Influenza Centre	Gregory, Vicki
EPI294210	A(H3N2)	HA	2017-12-20	A/Austria/1030866/2017	University of Vienna	Crick Worldwide Influenza Centre	Ermetal, Burcu
EPI294212	A(H3N2)	HA	2017-12-03	A/Baleares/2476/2017	Instituto de Salud Carlos III	Crick Worldwide Influenza Centre	Ermetal, Burcu
EPI294213	A(H3N2)	HA	2017-12-08	A/Brandenburg/45/2017	Robert Koch Institute Nationales Referenzzentrum f³r Influenza	Crick Worldwide Influenza Centre	Ermetal, Burcu
EPI294218	A(H3N2)	HA	2017-10-10	A/Finland/797/2017	National Institute for Health and Welfare	Crick Worldwide Influenza Centre	Ermetal, Burcu
EPI294219	A(H3N2)	HA	2017-11-09	A/Finland/798/2017	National Institute for Health and Welfare	Crick Worldwide Influenza Centre	Ermetal, Burcu
EPI294223	A(H3N2)	HA	2017-12-04	A/Galicia/2467/2017	Instituto de Salud Carlos III	Crick Worldwide Influenza Centre	Ermetal, Burcu
EPI294224	A(H3N2)	HA	2017-11-22	A/Ghana/832/2017	University of Ghana	Crick Worldwide Influenza Centre	Ermetal, Burcu
EPI294226	A(H3N2)	HA	2017-12-07	A/Hong Kong/4946/2017	Government Virus Unit	Crick Worldwide Influenza Centre	Ermetal, Burcu
EPI294230	A(H3N2)	HA	2017-12-18	A/Hong Kong/4995/2017	Government Virus Unit	Crick Worldwide Influenza Centre	Ermetal, Burcu
EPI294233	A(H3N2)	HA	2017-12-11	A/Navarra/2486/2017	Instituto de Salud Carlos III	Crick Worldwide Influenza Centre	Ermetal, Burcu
EPI294235	A(H3N2)	HA	2017-12-04	A/Nordrhein-Westfalen/132/2017	Robert Koch Institute Nationales Referenzzentrum f³r Influenza	Crick Worldwide Influenza Centre	Ermetal, Burcu
EPI294239	A(H3N2)	HA	2018-01-05	A/Northern Ireland/18000961/2018	Regional Virus Laboratory, Microbiology Department, Royal Victoria Ho:	Crick Worldwide Influenza Centre	Ermetal, Burcu
EPI294244	A(H3N2)	HA	2017-12-08	A/Norway/3784/2017	WHO National Influenza Centre	Crick Worldwide Influenza Centre	Ermetal, Burcu
EPI294246	A(H3N2)	HA	2017-10-06	A/Slovenia/2269/2017	Laboratory for Virology, National Institute of Public Health	Crick Worldwide Influenza Centre	Ermetal, Burcu
EPI294253	A(H3N2)	HA	2017-10-08	A/Valladolid/180/2017	Universidad de Valladolid	Crick Worldwide Influenza Centre	Ermetal, Burcu
EPI294255	A(H3N2)	HA	2017-10-24	A/Valladolid/182/2017	Universidad de Valladolid	Crick Worldwide Influenza Centre	Ermetal, Burcu
EPI294256	A(H3N2)	HA	2017-11-13	A/Zambia/305/2017	University Teaching Hospital	Crick Worldwide Influenza Centre	Ermetal, Burcu
EPI294258	A(H3N2)	HA	2017-11-30	A/Brandenburg/44/2017	Robert Koch Institute Nationales Referenzzentrum f³r Influenza	Crick Worldwide Influenza Centre	Ermetal, Burcu
EPI296168	A(H3N2)	HA	2016-06-14	A/Singapore/INFIMH-16-0019/2016	WHO Collaborating Centre for Reference and Research on Influenza	Centers for Disease Control and Prevention	DaSilva, Juliana
EPI297328	A(H3N2)	HA	2017-10-24	A/Banska Bystrica/33/2017	National Public Health Institute of Slovakia	Crick Worldwide Influenza Centre	Ermetal, Burcu
EPI297330	A(H3N2)	HA	2017-10-20	A/Croatia/3256/2017	Croatian Institute of Public Health	Crick Worldwide Influenza Centre	Ermetal, Burcu
EPI297333	A(H3N2)	HA	2018-01-02	A/Iceland/03/2018	Landspitali - University Hospital	Crick Worldwide Influenza Centre	Ermetal, Burcu
EPI297335	A(H3N2)	HA	2017-12-19	A/Iceland/133/2017	Landspitali - University Hospital	Crick Worldwide Influenza Centre	Ermetal, Burcu
EPI297336	A(H3N2)	HA	2017-12-27	A/Iceland/135/2017	Landspitali - University Hospital	Crick Worldwide Influenza Centre	Ermetal, Burcu
EPI297337	A(H3N2)	HA	2017-12-27	A/Iceland/136/2017	Landspitali - University Hospital	Crick Worldwide Influenza Centre	Ermetal, Burcu
EPI297345	A(H3N2)	HA	2018-01-01	A/Israel/R25/2018	Central Virology Laboratory Israel (NIC)	Crick Worldwide Influenza Centre	Ermetal, Burcu
EPI297346	A(H3N2)	HA	2018-01-02	A/Israel/R80/2018	Central Virology Laboratory Israel (NIC)	Crick Worldwide Influenza Centre	Ermetal, Burcu
EPI297347	A(H3N2)	HA	2017-12-24	A/Israel/SK521/2017	Central Virology Laboratory Israel (NIC)	Crick Worldwide Influenza Centre	Ermetal, Burcu
EPI297348	A(H3N2)	HA	2017-11-17	A/Lisboa/niSU024_17-18/2017	Instituto Nacional de Saude (INSA)	Crick Worldwide Influenza Centre	Ermetal, Burcu
EPI297351	A(H3N2)	HA	2017-12-27	A/Portugal/SU113/2017	Instituto Nacional de Saude (INSA)	Crick Worldwide Influenza Centre	Ermetal, Burcu
EPI297717	A(H3N2)	HA	2017-11-08	A/Astrakhan/32/2017		WHO National Influenza Centre Russian Federation	Fadeev, Artem
EPI297718	A(H3N2)	HA	2017-11-15	A/Saint-Petersburg/RII-330/2017		WHO National Influenza Centre Russian Federation	Fadeev, Artem
EPI297807	A(H3N2)	HA	2017-12-29	A/Slovenia/2792/2017	National Laboratory for Health Environment and Food	National Laboratory for Health Environment and Food	Berginc, Natasa
EPI303951	A(H3N2)	HA	2017-12-21	A/Switzerland/8060/2017	Hopital Cantonal Universitaire de Geneves	Crick Worldwide Influenza Centre	Ermetal, Burcu
EPI304556	A(H3N2)	HA	2017-11-22	A/Catalonia/3516779NS/2017	Barcelona, Facultad de Medicina	Crick Worldwide Influenza Centre	Ermetal, Burcu
EPI293829	B Yamagata	HA	2017-11-06	B/Perth/62/2017	Pathwest QE II Medical Centre	WHO Collaborating Centre for Reference and Research on Influenza	Komadina, Naomi
EPI132268	B Yamagata	HA	2012-08-03	B/Johannesburg/3964/2012	Sandringham National Institute for Communicable Diseases	National Institute for Medical Research	Gregory, Vicki
EPI208671	B Yamagata	HA	2016-01-04	B/Nordrhein-Westfalen/1/2016	Robert Koch Institute Nationales Referenzzentrum f³r Influenza	Crick Worldwide Influenza Centre	Gregory, Vicki
EPI208680	B Yamagata	HA	2016-01-14	B/Ireland/3154/2016	National Virus Reference Laboratory	Crick Worldwide Influenza Centre	Gregory, Vicki
EPI219327	B Yamagata	HA	2012-11-28	B/South Australia/81/2012	Institute of Medical and Veterinary Science (IMVS)	WHO Collaborating Centre for Reference and Research on Influenza	Komadina, Naomi
EPI246494	B Yamagata	HA	2008-08-04	B/Brisbane/60/2008	WHO Center for Reference & Research on Influenza	Crick Worldwide Influenza Centre	Ermetal, Burcu
EPI246895	B Yamagata	HA	2012-08-06	B/Formosa/V2367/2012	Instituto Nacional de Enfermedades Infecciosas	Crick Worldwide Influenza Centre	Ermetal, Burcu
EPI273908	B Yamagata	HA	2017-04-19	B/Moscow/114/2017	Ivanovsky Research Institute of Virology RAMS	Crick Worldwide Influenza Centre	Gregory, Vicki
EPI273915	B Yamagata	HA	2017-04-10	B/Novosibirsk/75/2017	WHO National Influenza Centre Russian Federation	Crick Worldwide Influenza Centre	Gregory, Vicki
EPI282641	B Yamagata	HA	2016-12-27	B/Maryland/15/2016	Centers for Disease Control and Prevention	WHO Collaborating Centre for Reference and Research on Influenza	Komadina, Naomi
EPI287082	B Yamagata	HA	2017-02-07	B/Niger/4582/2017	Centre de Recherche Medicale et Sanitaire (CERMES)	Crick Worldwide Influenza Centre	Gregory, Vicki
EPI287092	B Yamagata	HA	2017-02-08	B/Niger/4661/2017	Centre de Recherche Medicale et Sanitaire (CERMES)	Crick Worldwide Influenza Centre	Gregory, Vicki
EPI287093	B Yamagata	HA	2017-02-24	B/Niger/4674/2017	Centre de Recherche Medicale et Sanitaire (CERMES)	Crick Worldwide Influenza Centre	Gregory, Vicki
EPI289184	B Yamagata	HA	2017-11-08	B/Rancagua/90687/2017	Instituto de Salud Publica de Chile	Instituto de Salud Publica de Chile	Fernandez, Jorge Osvaldo
EPI291286	B Yamagata	HA	2017	B/Norway/2409/2017	Crick Worldwide Influenza Centre	WHO Collaborating Centre for Reference and Research on Influenza	Komadina, Naomi
EPI291527	B Yamagata	HA	2017-11-27	B/Centre/1582/2017	Institut Pasteur	Crick Worldwide Influenza Centre	Gregory, Vicki
EPI291528	B Yamagata	HA	2017-06-07	B/Norway/2957/2017	WHO National Influenza Centre	Crick Worldwide Influenza Centre	Gregory, Vicki
EPI291551	B Yamagata	HA	2017-09-12	B/Clermont-Ferrand/1894/2017	CNR Influenza France Sud	Crick Worldwide Influenza Centre	Gregory, Vicki
EPI291583	B Yamagata	HA	2017-11-08	B/Mexico/2735/2017	Laboratorio de Virus Respiratorio	Centers for Disease Control and Prevention	DaSilva, Juliana
EPI291615	B Yamagata	HA	2017-12-24	B/California/88/2017	California Department of Health Services	Centers for Disease Control and Prevention	DaSilva, Juliana
EPI292757	B Yamagata	HA	2017-03-24	B/Iwate/34/2017	National Institute of Infectious Diseases (NIID)	Centers for Disease Control and Prevention	DaSilva, Juliana
EPI293828	B Yamagata	HA	2017-10-20	B/Darwin/108/2017	Royal Darwin Hospital	WHO Collaborating Centre for Reference and Research on Influenza	Komadina, Naomi
EPI294967	B Yamagata	HA	2017-12-04	B/CastillaLaMancha/2439/2017	Instituto de Salud Carlos III	Crick Worldwide Influenza Centre	Gregory, Vicki
EPI294970	B Yamagata	HA	2017-11-17	B/Galicia/2407/2017	Instituto de Salud Carlos III	Crick Worldwide Influenza Centre	Gregory, Vicki
EPI294971	B Yamagata	HA	2017-12-02	B/Galicia/2465/2017	Instituto de Salud Carlos III	Crick Worldwide Influenza Centre	Gregory, Vicki
EPI294980	B Yamagata	HA	2017-12-19	B/Hong Kong/1095/2017	Government Virus Unit	Crick Worldwide Influenza Centre	Gregory, Vicki
EPI294985	B Yamagata	HA	2017-12-17	B/Hong Kong/1111/2017	Government Virus Unit	Crick Worldwide Influenza Centre	Gregory, Vicki
EPI294986	B Yamagata	HA	2017-12-17	B/Hong Kong/1118/2017	Government Virus Unit	Crick Worldwide Influenza Centre	Gregory, Vicki

Isolate ID	Type	Segment	Collection date	Isolate Name	Originating Laboratory	Submitting Laboratory	Authors
EPI294991	B Yamagata	HA	2017-11-29	B/Hong Kong/930/2017	Government Virus Unit	Crick Worldwide Influenza Centre	Gregory, Vicki
EPI295009	B Yamagata	HA	2017-12-12	B/Slovenia/2654/2017	Laboratory for Virology, National Institute of Public Health	Crick Worldwide Influenza Centre	Gregory, Vicki
EPI295013	B Yamagata	HA	2017-12-04	B/Spain/107178/2017	Universidad de Valladolid	Crick Worldwide Influenza Centre	Gregory, Vicki
EPI295014	B Yamagata	HA	2017-12-07	B/Spain/108127/2017	Universidad de Valladolid	Crick Worldwide Influenza Centre	Gregory, Vicki
EPI296170	B Yamagata	HA	2017-11-15	B/Buenos Aires/11926546/2017	Instituto Nacional de Enfermedades Infecciosas	Crick Worldwide Influenza Centre	Ermetal, Burcu
EPI296184	B Yamagata	HA	2017-12-29	B/Portugal/SU144/2017	Instituto Nacional de Saude (INSA)	Crick Worldwide Influenza Centre	Ermetal, Burcu
EPI296187	B Yamagata	HA	2017-12-28	B/Slovenia/2786/2017	Laboratory for Virology, National Institute of Public Health	Crick Worldwide Influenza Centre	Ermetal, Burcu
EPI296190	B Yamagata	HA	2017-11-13	B/Valladolid/183/2017	Universidad de Valladolid	Crick Worldwide Influenza Centre	Ermetal, Burcu
EPI296373	B Yamagata	HA	2017-11-06	B/Alberta/RV2559/2017	National Microbiology Laboratory Health Canada	Centers for Disease Control and Prevention	DaSilva, Juliana
EPI297023	B Yamagata	HA	2018-01-02	B/Yunnan-Wenshan/11/2018	WHO Chinese National Influenza Center	WHO Chinese National Influenza Center	Zeng, Xiaoxu
EPI297024	B Yamagata	HA	2017-11-21	B/Hubei-Zhangwan/36/2017	WHO Chinese National Influenza Center	WHO Chinese National Influenza Center	Zeng, Xiaoxu
EPI299705	B Yamagata	HA	2017-05-26	B/Laos/F1664/2017	National Institute of Infectious Diseases (NIID)	WHO Collaborating Centre for Reference and Research on Influenza	Komadina, Naomi
EPI30253	B Yamagata	HA	2004	B/Malaysia/2506/2004		Other Database Import	Initial import ...
EPI303188	B Yamagata	HA	2017-12-04	B/Hubei-Jiangan/1974/2017	WHO Chinese National Influenza Center	Centers for Disease Control and Prevention	DaSilva, Juliana
EPI70224	B Yamagata	HA	2009-10-11	B/Hong Kong/514/2009	Government Virus Unit	National Institute for Medical Research	Gregory, Vicki
EPI99942	B Yamagata	HA	2011-03-07	B/Malta/MV636714/2011	Mater Dei Hospital	National Institute for Medical Research	Gregory, Vicki
EPI293829	B Victoria	HA	2017-11-06	B/Perth/62/2017	Pathwest QE II Medical Centre	WHO Collaborating Centre for Reference and Research on Influenza	Komadina, Naomi
EPI132268	B Victoria	HA	2012-08-03	B/Johannesburg/3964/2012	Sandringham National Institute for Communicable Diseases	National Institute for Medical Research	Gregory, Vicki
EPI208671	B Victoria	HA	2016-01-04	B/Nordrhein-Westfalen/1/2016	Robert Koch Institute Nationales Referenzzentrum f³r Influenza	Crick Worldwide Influenza Centre	Gregory, Vicki
EPI208680	B Victoria	HA	2016-01-14	B/Ireland/3154/2016	National Virus Reference Laboratory	Crick Worldwide Influenza Centre	Gregory, Vicki
EPI219327	B Victoria	HA	2012-11-28	B/South Australia/81/2012	Institute of Medical and Veterinary Science (IMVS)	WHO Collaborating Centre for Reference and Research on Influenza	Komadina, Naomi
EPI246494	B Victoria	HA	2008-08-04	B/Brisbane/60/2008	WHO Centre for Reference & Research on Influenza	Crick Worldwide Influenza Centre	Ermetal, Burcu
EPI246895	B Victoria	HA	2012-08-06	B/Formosa/V2367/2012	Instituto Nacional de Enfermedades Infecciosas	Crick Worldwide Influenza Centre	Ermetal, Burcu
EPI273908	B Victoria	HA	2017-04-19	B/Moscow/114/2017	Ivanovsky Research Institute of Virology RAMS	Crick Worldwide Influenza Centre	Gregory, Vicki
EPI273915	B Victoria	HA	2017-04-10	B/Novosibirsk/75/2017	WHO National Influenza Centre Russian Federation	Crick Worldwide Influenza Centre	Gregory, Vicki
EPI282641	B Victoria	HA	2016-12-27	B/Maryland/15/2016	Centers for Disease Control and Prevention	WHO Collaborating Centre for Reference and Research on Influenza	Komadina, Naomi
EPI287082	B Victoria	HA	2017-02-07	B/Niger/4582/2017	Centre de Recherche Medicale et Sanitaire (CERMES)	Crick Worldwide Influenza Centre	Gregory, Vicki
EPI287092	B Victoria	HA	2017-02-08	B/Niger/4661/2017	Centre de Recherche Medicale et Sanitaire (CERMES)	Crick Worldwide Influenza Centre	Gregory, Vicki
EPI287093	B Victoria	HA	2017-02-24	B/Niger/4674/2017	Centre de Recherche Medicale et Sanitaire (CERMES)	Crick Worldwide Influenza Centre	Gregory, Vicki
EPI289184	B Victoria	HA	2017-11-08	B/Rancagua/90687/2017	Instituto de Salud Publica de Chile	Instituto de Salud Publica de Chile	Fernandez, Jorge Osvaldo
EPI291286	B Victoria	HA	2017	B/Norway/2409/2017	Crick Worldwide Influenza Centre	WHO Collaborating Centre for Reference and Research on Influenza	Komadina, Naomi
EPI291527	B Victoria	HA	2017-11-27	B/Centre/1582/2017	Institut Pasteur	Crick Worldwide Influenza Centre	Gregory, Vicki
EPI291528	B Victoria	HA	2017-06-07	B/Norway/2957/2017	WHO National Influenza Centre	Crick Worldwide Influenza Centre	Gregory, Vicki
EPI291551	B Victoria	HA	2017-09-12	B/Clermont-Ferrand/1894/2017	CNR Influenza France Sud	Crick Worldwide Influenza Centre	Gregory, Vicki
EPI291583	B Victoria	HA	2017-11-08	B/Mexico/2735/2017	Laboratorio de Virus Respiratorio	Centers for Disease Control and Prevention	DaSilva, Juliana
EPI291615	B Victoria	HA	2017-12-24	B/California/88/2017	California Department of Health Services	Centers for Disease Control and Prevention	DaSilva, Juliana
EPI292757	B Victoria	HA	2017-03-24	B/Iwate/34/2017	National Institute of Infectious Diseases (NIID)	Centers for Disease Control and Prevention	DaSilva, Juliana
EPI293828	B Victoria	HA	2017-10-20	B/Darwin/108/2017	Royal Darwin Hospital	WHO Collaborating Centre for Reference and Research on Influenza	Komadina, Naomi
EPI294967	B Victoria	HA	2017-12-04	B/CastillaLaMancha/2439/2017	Instituto de Salud Carlos III	Crick Worldwide Influenza Centre	Gregory, Vicki
EPI294970	B Victoria	HA	2017-11-17	B/Galicia/2407/2017	Instituto de Salud Carlos III	Crick Worldwide Influenza Centre	Gregory, Vicki
EPI294971	B Victoria	HA	2017-12-02	B/Galicia/2465/2017	Instituto de Salud Carlos III	Crick Worldwide Influenza Centre	Gregory, Vicki
EPI294980	B Victoria	HA	2017-12-19	B/Hong Kong/1095/2017	Government Virus Unit	Crick Worldwide Influenza Centre	Gregory, Vicki
EPI294985	B Victoria	HA	2017-12-17	B/Hong Kong/1111/2017	Government Virus Unit	Crick Worldwide Influenza Centre	Gregory, Vicki
EPI294986	B Victoria	HA	2017-12-17	B/Hong Kong/1118/2017	Government Virus Unit	Crick Worldwide Influenza Centre	Gregory, Vicki
EPI294991	B Victoria	HA	2017-11-29	B/Hong Kong/930/2017	Government Virus Unit	Crick Worldwide Influenza Centre	Gregory, Vicki
EPI295009	B Victoria	HA	2017-12-12	B/Slovenia/2654/2017	Laboratory for Virology National Institute of Public Health	Crick Worldwide Influenza Centre	Gregory, Vicki
EPI295013	B Victoria	HA	2017-12-04	B/Spain/107178/2017	Universidad de Valladolid	Crick Worldwide Influenza Centre	Gregory, Vicki
EPI295014	B Victoria	HA	2017-12-07	B/Spain/108127/2017	Universidad de Valladolid	Crick Worldwide Influenza Centre	Gregory, Vicki
EPI296170	B Victoria	HA	2017-11-15	B/Buenos Aires/11926546/2017	Instituto Nacional de Enfermedades Infecciosas	Crick Worldwide Influenza Centre	Ermetal, Burcu
EPI296184	B Victoria	HA	2017-12-29	B/Portugal/SU144/2017	Instituto Nacional de Saude (INSA)	Crick Worldwide Influenza Centre	Ermetal, Burcu
EPI296187	B Victoria	HA	2017-12-28	B/Slovenia/2786/2017	Laboratory for Virology	National Institute of Public Health	Crick Worldwide Influenza Centre
EPI296190	B Victoria	HA	2017-11-13	B/Valladolid/183/2017	Universidad de Valladolid	Crick Worldwide Influenza Centre	Ermetal, Burcu
EPI296373	B Victoria	HA	2017-11-06	B/Alberta/RV2559/2017	National Microbiology Laboratory Health Canada	Centers for Disease Control and Prevention	DaSilva, Juliana
EPI297023	B Victoria	HA	2018-01-02	B/Yunnan-Wenshan/11/2018	WHO Chinese National Influenza Center	WHO Chinese National Influenza Center	Zeng, Xiaoxu
EPI297024	B Victoria	HA	2017-11-21	B/Hubei-Zhangwan/36/2017	WHO Chinese National Influenza Center	WHO Chinese National Influenza Center	Zeng, Xiaoxu
EPI299705	B Victoria	HA	2017-05-26	B/Laos/F1664/2017	National Institute of Infectious Diseases (NIID)	WHO Collaborating Centre for Reference and Research on Influenza	Komadina, Naomi
EPI30253	B Victoria	HA	2004	B/Malaysia/2506/2004		Other Database Import	Initial import ...
EPI303188	B Victoria	HA	2017-12-04	B/Hubei-Jiangan/1974/2017	WHO Chinese National Influenza Center	Centers for Disease Control and Prevention	DaSilva, Juliana
EPI70224	B Victoria	HA	2009-10-11	B/Hong Kong/514/2009	Government Virus Unit	National Institute for Medical Research	Gregory, Vicki
EPI99942	B Victoria	HA	2011-03-07	B/Malta/MV636714/2011	Mater Dei Hospital	National Institute for Medical Research	Gregory, Vicki

(1) Shu, Y., McCauley, J. (2017) GISAID: Global initiative on sharing all influenza data – from vision to reality
EuroSurveillance, 22(13) doi:10.2807/1560-7917.ES.2017.22.13.30494 PMCID: PMC5388101
